# The *EIL* transcription factor family in soybean: Genome‐wide identification, expression profiling and genetic diversity analysis

**DOI:** 10.1002/2211-5463.12596

**Published:** 2019-02-21

**Authors:** Qing Li, Yanting Shen, Luqin Guo, Hong Wang, Yu Zhang, Chengming Fan, Yihong Zheng

**Affiliations:** ^1^ College of Life Sciences and Oceanography Shenzhen University China; ^2^ Key Laboratory of Optoelectronic Devices and Systems of Ministry of Education and Guangdong Province College of Optoelectronic Engineering Shenzhen University China; ^3^ Institute of Genetics and Developmental Biology Chinese Academy of Sciences Beijing China; ^4^ College of Horticulture Henan Agricultural University Zhengzhou China

**Keywords:** *EIL* gene family, ethylene, ethylene signaling, soybean

## Abstract

The *ETHYLENE INSENSITIVE3‐LIKE (EIL)* transcription factor family plays a critical role in the ethylene signaling pathway, which regulates a broad spectrum of plant growth and developmental processes, as well as defenses to myriad stresses. Although genome‐wide analysis of this family has been carried out for several plant species, no comprehensive analysis of the *EIL* gene family in soybean has been reported so far. Furthermore, there are few studies on the functions of *EIL* genes in soybean. In this study, we identified 12 soybean (*Gm*) *EIL* genes, which we divided into three groups based on their phylogenetic relationships. We then detected their duplication status and found that most of the *GmEIL* genes have duplicated copies derived from two whole‐genome duplication events. These duplicated genes underwent strong negative selection during evolution. We further analyzed the transcript profiles of *GmEIL* genes using the transcriptome data and found that their spatio‐temporal and stress expression patterns varied considerably. For example, *GmEIL1*–*GmEIL5* were found to be strongly expressed in almost every sample, while *GmEIL8*–*GmEIL12* exhibited low expression, or were not expressed at all. Additionally, these genes showed different responses to dehydration, salinity and phosphate starvation. Finally, we surveyed genetic variations of these genes in 302 resequenced wild soybeans, landraces and improved soybean cultivars. Our data showed that most *GmEIL* genes are well conserved, and are not modified in domesticated or improved cultivars. Together, these findings provide a potentially valuable resource for characterizing the *GmEIL* gene family and lay the basis for further elucidation of their molecular mechanisms.

**Abbreviations**
EIL: EIN3‐LIKE
EIN: ETHYLENE INSENSITIVE
FC: fold‐change
FPKM: fragments per kilobase of exon per million fragments mapped
*K*_a_: non‐synonymous substitution rate
*K*_s_: synonymous substitution rate
P_i_: phosphate
SNP: single nucleotide polymorphism
TF: transcription factor
WGD: whole‐genome duplication

Ethylene, the gaseous and smallest phytohormone with a simple C_2_H_4_ structure, regulates a number of developmental processes, including cell division and expansion, seed germination, root initiation, leaf growth, flower development, sex determination, fruit ripening, and organ senescence 1, 2. In addition, it also has multiple functions in stress defenses, as it is produced in response to both biotic and abiotic challenges, such as flooding, wounding, heat, cold, low nutrition, salt stress and pathogen attack 2, 3, 4. During the past decades, a series of important ethylene signaling components have been identified through the application of molecular and genetic approaches, and the core ethylene signaling pathway has been well established 1, 4. In the model plant Arabidopsis, ethylene triggers a signaling cascade initiated by a group of ER‐located receptors [ETHYLENE RESISTANCE (ETR) 1, ETR2, ETHYLENE RESPONSE SENSOR (ERS) 1, ERS2 and ETHYLENE INSENSITIVE (EIN) 4] 5. These receptors are inactive in the presence of ethylene, which otherwise represses ethylene responses through binding to and thereby activating the negative regulator CONSTITUTIVE TRIPLE RESPONSE 1 (CTR1) 6, 7, 8, 9. CTR1 is a kinase that represses the ER‐located EIN2 by protein phosphorylation in the absence of ethylene 10. When this inhibition is relieved, EIN2 is dephosphorylated and cleaved, releasing a functional C‐terminal fragment that moves either to P‐bodies or to the nucleus 11, 12, 13, 14. The EIN2 activation triggers the stabilization of EIN3 and its homolog EIN3‐LIKE (EIL) 1, which function as primary transcription factors (TFs) in the ethylene signaling pathway and further initiate a transcriptional cascade involving ETHYLENE RESPONSE FACTORs 4, 15, 16.

*EIN3*,* EIL1* and four other members (*EIL*2 to *EIL5*) constitute the *EIL* gene family in Arabidopsis, which encode a small class of plant‐specific TFs possessing highly acidic, basic and proline‐rich domains 17, 18. Among these TFs, EIN3 and its closest homolog EIL1 play the major but partially redundant roles in the ethylene signaling pathway, whereas the less homologous members (EIL2 to EIL5) in the EIL family might either have minor effects in the ethylene responses in specific tissues and developmental stages or function in completely different pathways that are unrelated to ethylene responses 17. For example, the overexpression of *EIN3* or *EIL1* confers a constitutive ethylene phenotype in wild‐type plants or the *ein2* mutants, and their single mutants, both *ein3* and *eil1*, show partial ethylene insensitivity, but *ein3 eil1* double mutants display completely ethylene‐insensitive phenotypes in all known ethylene responses 17, 18. Furthermore, overexpression of *EIL*2 can rescue both the seedling and adult *ein3‐1* mutant phenotypes, although it does not naturally complement the *ein3* mutation due to its lower expression level than that of *EIN3* or *EIL1* 18. In contrast, EIL3 (also named SLIM1) is a central transcriptional regulator of plant sulfur response and metabolism. Overexpression of *EIL3*, but not other *EIL* genes of Arabidopsis, restores the sulfur limitation responseless phenotypes of *slim1* mutants 19. A recent report suggests a potential crosstalk between sulfur assimilation and ethylene signaling pathways via a direct EIN3–EIL3 interaction 20. In addition to directly regulating the ethylene signaling pathway, the EIN3/EIL1 TFs also act as a hub for ethylene connections with other signals, such as the crosstalk between ethylene and other hormones, light signaling, as well as various abiotic and biotic stress responses 4, 16.

To date, our knowledge about the *EIL* TF family has mainly been obtained from the model plant Arabidopsis, although the functions of these genes have been studied in several other plants, such as rice 19, 21, 22, 23, tomato 24, 25, tobacco 26, 27 and cucumber 28. The regulation mechanisms of the *EIL* TF family in soybean, an important crop for seed protein and oil content, are remain poorly understood. Thus, genome‐wide identification and analysis of the *EIL* TF family would be essential to elucidate the roles of ethylene signaling in soybean. In this work, a systematic analysis was performed to study the *EIL* TF family in soybean. A total of 12 soybean (*Gm*) *EIL* genes were identified and categorized based on their characteristics for phylogenetic relationships, gene structures and motif compositions. We further surveyed their duplication status, spatio‐temporal and stressed expression patterns as well as genetic diversity. Our results provide a framework for the future functional study of *GmEIL* genes. Furthermore, this study may also contribute to knowledge of the ethylene signaling pathway in soybean.

## Materials and methods

### Identification of *EIL* TF family members

To identify *EIL* TF family members in soybean, the sequences of Arabidopsis AtEIN3 and AtEIL1–AtEIL5 proteins were used as query to search the soybean genome in Phytozome (https://phytozome.jgi.doe.gov//portal.html). Then, the Pfam tool (http://pfam.xfam.org/) was used to verify the retrieved GmEIL candidates with the typical EIN3 domain 29. Similarly, the *EIL* TF family members of 18 representative species were screened from their respective genome. The genome sequences of soybean and 18 representative species were used to generate a phylogenetic tree using the phylot tool (https://phylot.biobyte.de/).

### Phylogenetic analysis and characterization of *EIL* TF family

The full amino acid sequences of EIL members from Arabidopsis and soybean were aligned by the ClustalW method. Then, a neighbor‐joining phylogenetic tree was constructed using mega 6.0 (https://www.megasoftware.net/) with a Poisson model and 1000 bootstraps 30. The *EIL* gene structures were drawn with gsds 2.0 software (http://gsds.cbi.pku.edu.cn/) based on their genomic DNA annotations 31. The molecular masses and isoelectric points of EIL proteins were acquired from ProtParam tool (https://web.expasy.org/protparam/). The subcellular localizations of EIL proteins were analyzed using the Plant‐mPLoc server (http://www.csbio.sjtu.edu.cn/bioinf/plant-multi/) 32. The 10 conserved motifs of EIL proteins were identified by meme (http://meme-suite.org/) 33. The *cis*‐acting regulatory elements in each promoter (1.5 kb upstream of the ATG starting site) of *EIL* genes were predicated using the PlantCARE database (http://bioinformatics.psb.ugent.be/webtools/plantcare/html/).

### Identification of syntenic blocks of the *GmEIL* TF family

The syntenic blocks containing *GmEIL* genes in soybean were identified using the mcscanx toolkit 34. Briefly, blastp with *e*‐value < 1e‐5 was employed to search the best five homologs in the genome. The acquired blastp results were next used as the mcscanx input to assess the collinear blocks. The collinear relationships of *GmEIL* genes were painted with circos software 35. The non‐synonymous (*K*_a_) and synonymous (*K*_s_) substitution rates between paralog pairs were determined by dnasp (version 6) 36.

### RNA‐seq and data analysis

Our previously published Illumina (San Diego, CA, USA) RNA‐seq data for 28 samples with various tissues and developmental stages were used to detect the spatio‐temporal expression patterns of *GmEIL* genes 37. The raw reads were mapped to the soybean reference genome Wm82.a2.v1 utilizing hisat 38. The transcripts assembly and expression counts were gained using stringtie 39. The fragments per kilobase of exon per million fragments mapped (FPKM) value was used to represent the gene expression value. To investigate the expression profiles of *GmEIL* genes against abiotic stresses, we explored them using previously reported Illumina RNA‐seq data regarding dehydration, salt stress and phosphate (P_i_) starvation 40, 41, 42. Similarly, the gene expression value was also calculated by FPKM. Differential expression was carried out by comparing the expression of a gene in each sample to control. Both the FPKM and fold‐change (FC) values were log2 transformed and exhibited in the form of heat maps using the heml tool 43.

### SNP genotyping of the *GmEIL* TF family

The single nucleotide polymorphisms (SNPs) of *GmEIL* genes in 302 soybean accessions were extracted from our released whole‐genome resequencing data 44. Read mapping and SNP calling were executed according to a previously described method 45. The genomic region was divided into 5′‐untranslated region (UTR), exon, intron and 3′‐UTR based on the genome annotation. The SNPs were classified as synonymous SNPs (no amino acid change), non‐synonymous SNPs (cause amino acid substitutions) and premature SNPs (generate a stop codon).

## Results

### Genome‐wide identification of *GmEIL* TF family members

To identify the *GmEIL* genes, the Arabidopsis EIL family amino acid sequences were used as query to perform a genome‐wide search in soybean. The major domains of the retrieved GmEIL candidates were further detected by Pfam (Fig. S1). By discarding the non‐primary transcripts of the same gene, a total of 12 *EIL* TF family members with the conserved EIN3 domain were identified in soybean. For convenience, we named them *GmEIL1* to *GmEIL12* in order (Table 1). The 12 *GmEIL* genes are distributed across 10 of the 20 chromosomes in the soybean genome. Among them, chromosome 13 has three *GmEIL* genes, whereas chromosomes 2, 5, 6, 8, 11, 14, 15, 18 and 20 only contain one *GmEIL* gene, and no *GmEIL* gene is located on the remaining chromosomes (Table 1). The amino acid lengths of the 12 GmEIL proteins range from 398 to 766, the molecular masses extend from 45 263.15 to 84 847.37 Da, and their inferred isoelectric points range from 4.88 to 5.82 (Table 1). These proteins vary greatly from 32.4% to 96.2% in sequence identity (Fig. S3), although the amino‐terminal halves of these polypeptides are more conserved than their carboxy‐terminal regions (Fig. S2). All of these GmEIL proteins, as Arabidopsis EIL family proteins, were inferred to be localized in the nucleus, which is consistent with their function as TFs (Table 1).

**Table 1:** *EIL* genes in Arabidopsis and soybean. The physical position of each *EIL* gene is indicated and ‘+’ and ‘−’ indicate the genes are forward and reverse in the genome, respectively. Protein length is shown as number of amino acids. p*I* is the theoretical isoelectric point

Gene name	Gene ID	Gene localization	Protein			
			Length (aa)	*M* (Da)	p*I*	Localization
*AtEIN3*	AT3G20770.1	Chr03:7260432–7263352 −	628	71 421.41	5.62	Nucleus
*AtEIL1*	AT2G27050.1	Chr02:11545753–11548293 +	584	66 495.44	5.83	Nucleus
*AtEIL2*	AT5G21120.1	Chr05:7182621–7184342 +	518	59 185.71	5.75	Nucleus
*AtEIL3*	AT1G73730.1	Chr01:27730031–27732514 −	567	64 041.53	5.28	Nucleus
*AtEIL4*	AT5G10120.1	Chr05:3169732–3171147 +	471	53 954.14	5.30	Nucleus
*AtEIL5*	AT5G65100.1	Chr05:26006835–26008508 −	557	63 689.59	4.77	Nucleus
*GmEIL1*	Glyma.20G051500.1	Chr20:11509210–11512726 −	624	70 651.75	5.51	Nucleus
*GmEIL2*	Glyma.13G076700.1	Chr13:18122954–18126172 −	621	70 451.49	5.33	Nucleus
*GmEIL3*	Glyma.14G041500.1	Chr14:3127834–3131281 +	610	69 010.99	5.45	Nucleus
*GmEIL4*	Glyma.02G274600.1	Chr02:45772152–45775672 −	614	69 589.49	5.49	Nucleus
*GmEIL5*	Glyma.13G076800.1	Chr13:18149791–18153194 +	618	70 088.24	5.51	Nucleus
*GmEIL6*	Glyma.13G342500.1	Chr13:43396036–43399003 −	591	66 052.93	5.77	Nucleus
*GmEIL7*	Glyma.15G031800.1	Chr15:2560344–2563456 +	590	66 129.09	5.75	Nucleus
*GmEIL8*	Glyma.08G137800.1	Chr08:10565832–10577477 +	453	52 092.6	5.03	Nucleus
*GmEIL9*	Glyma.05G180300.1	Chr05:36841419–36842807 +	462	53 133.77	5.10	Nucleus
*GmEIL10*	Glyma.06G314000.1	Chr06:50290254–50293347 −	766	84 847.37	5.82	Nucleus
*GmEIL11*	Glyma.18G018400.1	Chr18:1345938–1348252 +	464	52 506.33	4.88	Nucleus
*GmEIL12*	Glyma.11G239000.1	Chr11:33337797–33339122 −	398	45 263.15	5.08	Nucleus

**Figure 1:**
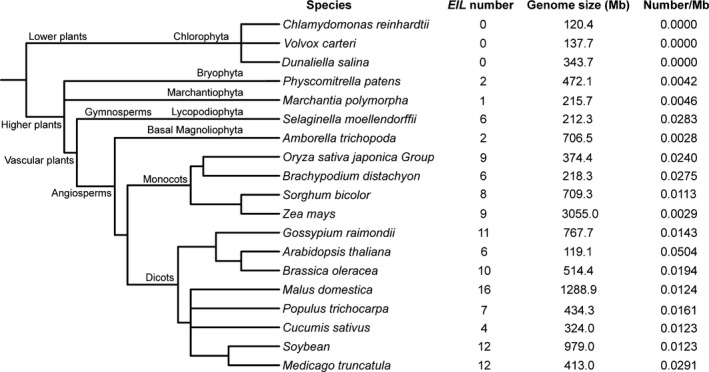
Summary of the *EIL* TF family in soybean and 18 representative species.

To perform comparative genomic analysis, we searched for the *EIL* TF family in the genomes of 18 other representative species, including seven dicots, four monocots, one basal angiosperm, one gymnosperm, one bryophyte, one marchantiophyte and three chlorophytes. After filtering, a total of 109 *EIL* genes were identified among all these species (Fig. 1). In general, there were more *EIL* genes in monocots and dicots than in other higher plants, and no *EIL* gene was identified in the three chlorophytes (Fig. 1). This result indicates that the *EIL* genes were expanded after the divergence of the higher plants from the lower plants, and that they may play important roles during the evolution of the higher plants. In addition, we found the number of *EIL* genes is not positively correlated with the genome size and duplication event of species, which is also reflected by the density variations of *EIL* genes (Fig. 1). For example, *Zea mays* has the largest genome size, but both its *EIL* genes number and average density are fewer than those of *Gossypium raimondii*,* Medicago truncatula*,* Brassica oleracea* and *Malus domestica*. Similarly, not only the number but also the average density of *EIL* genes in paleopolyploid soybean are not more than those in diploid *M. truncatula*. This result implicates that the *EIL* TF family members have rapid and different evolution processes in various plants.

### Phylogenetic, gene structure and protein motif analysis of *GmEIL* genes

**Figure 2:**
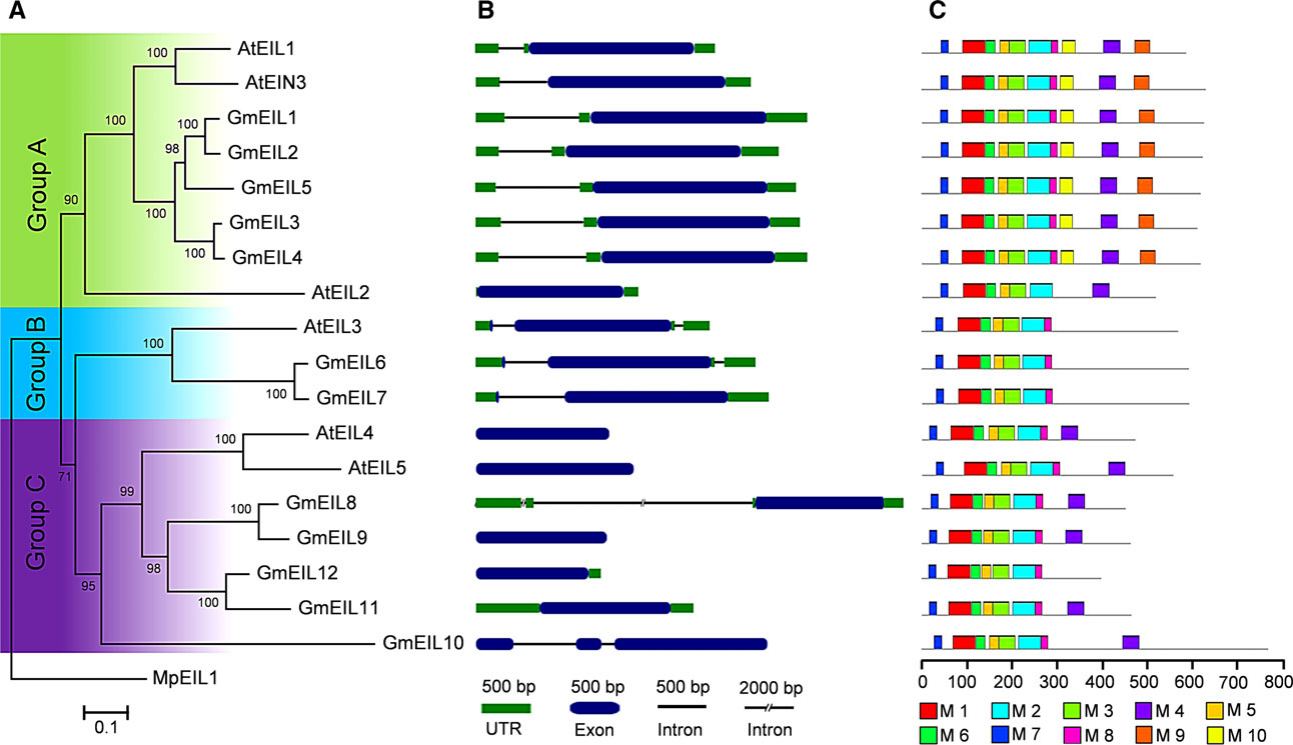
Phylogenetic relationships, gene structures and motif compositions of *EIL* genes from Arabidopsis and soybean. (A) The phylogenetic tree of EILs. A neighbor‐joining tree was constructed with mega 6.0 software using protein sequences. The *Marchantia polymorpha* EIL (Mapoly0088s0024.1) protein was used as an outgroup. (B) The exon–intron structures of *EILs*. Gene structural features were drawn using gsds 2.0 software. (C) The motif distribution of EILs. The conserved motifs were identified using the meme program. Different motifs are represented by different colored boxes numbered M1–M10.

To assess the phylogenetic relationships of *GmEIL* genes, we constructed a phylogenetic tree with the EIL family protein sequences from soybean and Arabidopsis (Fig. 2A). The result showed that 12 *GmEIL* genes were obviously classified into three groups (designated as A, B and C) based on the bootstrap values and phylogenetic topology. Group A contained five members (*GmEIL1* to *GmEIL5*), group B had two members (*GmEIL6* and *GmEIL7*), while group C contained the others (*GmEIL8* to *GmEIL12*).

Previous research suggested that the gene structural diversity among gene family members is a primary resource for the evolution of multiple gene families 46. To characterize the structural diversity of the *EIL* genes, their exon–intron organizations were analyzed according to the genomic DNA annotations (Fig. 2B). This showed that most *EIL* genes in the same group shared almost uniform exon–intron structures. For example, the *EIL* genes in group A contained one exon, whereas two exons were present in those genes in group B. All of the *EIL* genes in group C except *GmEIL10* also only had one exon.

Proteins that share common motif compositions in the same family are likely to have similar functions 33. Thus, the most conserved 10 motifs among the soybean and Arabidopsis EIL proteins were predicted by meme (Fig. 2C, Fig. S4). Remarkably, most of the closely related EIL proteins within the same group displayed similar motif compositions, indicating their functional similarities. For instance, all of the EIL proteins in group A except AtEIL2 had the motifs 1–10. Moreover, the EIL proteins in group C without GmEIL12 had the motifs 1–8. Compared with the EIL proteins of group C, motif 4 was lost in those proteins of group B.

Taken together, the similarities in gene structures and motif distributions of most EIL members support the results from phylogenetic analysis, and the differences of the related characteristics in the different groups implicate that they have divergent functions.

### Duplication status of the *GmEIL* genes within the soybean genome

**Figure 3:**
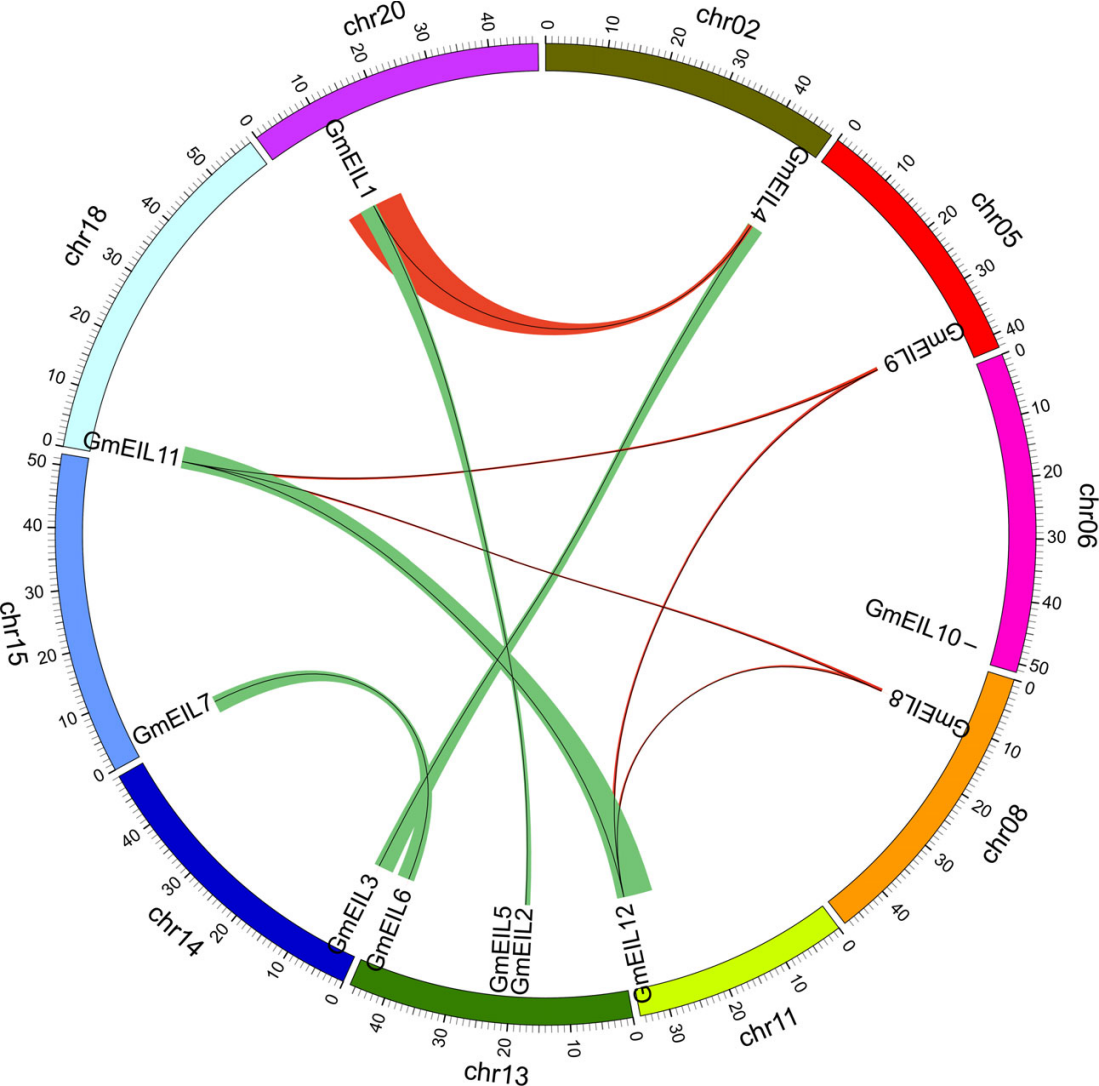
The collinear relationships of homologous blocks containing *GmEIL* genes. The green and red colored rainbows represent the collinear relationships that arose from the *Glycine* WGD event and legume WGD event, respectively. The black lines within these blocks display the location of *GmEIL* genes. The positions of *GmEIL2* and *GmEIL5* were hard to separate since they are adjacent in the same chromosome.

Soybean is a paleopolyploid plant that has experienced at least two rounds of whole‐genome duplication (WGD) events, leading to a highly duplicated soybean genome with approximately 75% of the genes existing in multiple copies 47. The existence of duplicated genes could provide more chances for gene evolution via neofunctionalization, subfunctionalization and non‐functionalization 48, 49. Therefore, it would be useful to detect the duplication status of *GmEIL* genes. Using a collinearity analysis, we found that all the *GmEIL* genes, apart from *GmEIL5* and *GmEIL10*, have duplicated copies generated from the two WGD events (Fig. 3). Among these duplicated genes, *GmEIL1* and *GmEIL2*,* GmEIL3* and *GmEIL4*,* GmEIL6* and *GmEIL7*, and *GmEIL11* and *GmEIL12* came from the recent *Glycine* WGD event at 13 million years ago because their *K*_s_ values are < 0.3. In contrast, the remaining duplicated genes were derived from the ancient legume WGD event at 59 million years ago since their *K*_s_ values are between 0.3 and 1.5 47. Besides the WGD duplication, *GmEIL5* may have experienced a tandem duplication event with *GmEIL2*, considering they are located next to each other in the same chromosome (Fig. 3).

In addition, the *K*_a_/*K*_s_ value is frequently used to represent the selection pressure and evolution rate of duplicated genes. As reported earlier, *K*_a_/*K*_s_ > 1 indicates positive selection with accelerated (diversifying) evolution, *K*_a_/*K*_s_ < 1 indicates negative (purifying) selection with a functional constraint, and *K*_a_/*K*_s_ = 1 indicates neutral mutation or no selection 50. In this work, all paralogs were found with *K*_a_/*K*_s_ values < 0.3 (Table S1), indicating their strongly negative selection during evolution. The highly evolutionary constraints in *GmEIL* genes may contribute to their functional stability.

### The spatio‐temporal expression profiles of *GmEIL* genes

**Figure 4:**
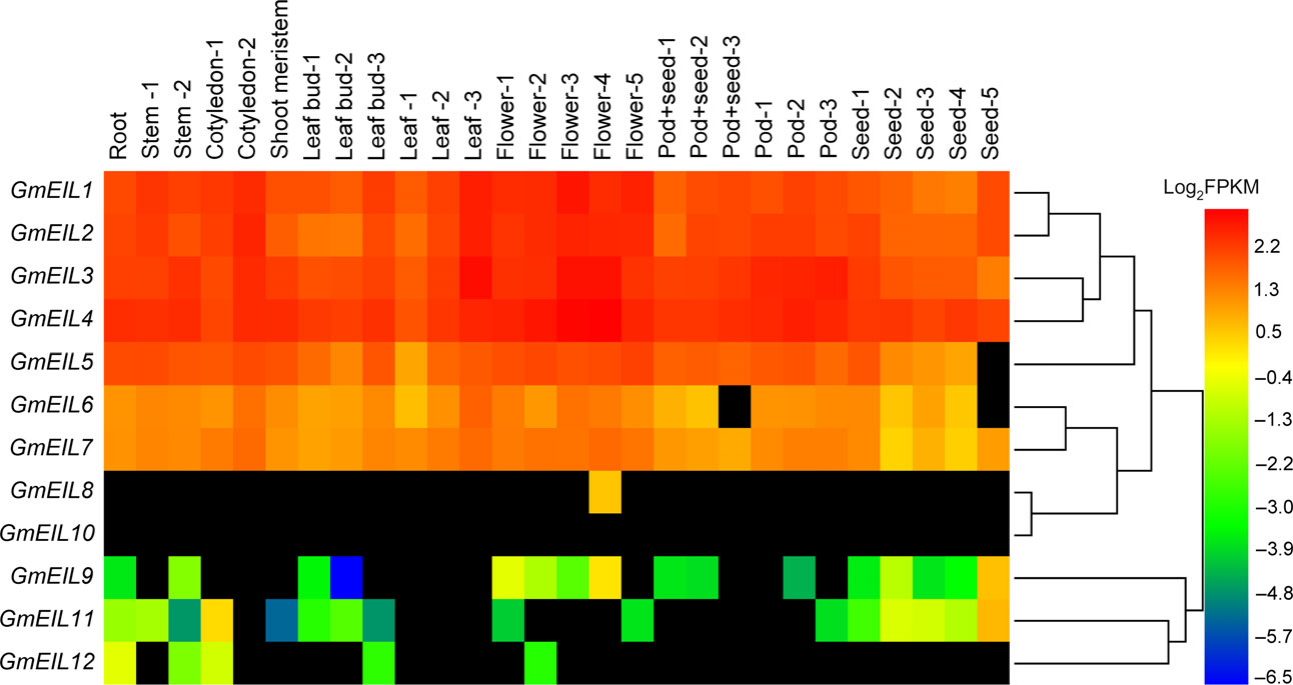
The spatio‐temporal expression profiles of *GmEIL* genes in soybean. The gene expression values (FPKM values) were log2 transformed and displayed in the form of heat maps. Black indicates an FPKM value of 0. The numbers near the same tissue/organ represent earlier to later developmental stages.

Gene expression pattern can provide important clues to gene function. To achieve the spatio‐temporal expression profiles of *GmEIL* genes in soybean, we explored those in 28 samples by using our previously published Illumina RNA‐seq data 37. The results suggested that the expression levels and patterns of these genes in different groups varied considerably (Fig. 4). In detail, the *GmEIL* genes in group A showed uniformly high expression in almost every sample. In contrast, these *GmEIL* genes from group C exhibited significantly lower or no expression in most tissues. Among them, *GmEIL8* displayed tissue‐specific expression, which was only detected in flower at stage 4 (5 days after flowering). *GmEIL10* was a potential pseudogene due to its no or extremely low expression values in all samples. Additionally, *GmEIL6* and *GmEIL7* in group B exhibited intermediate expression levels in most tissues compared with those genes from groups A and C. Taken together, the differential expression patterns of *GmEIL* genes, especially for those in different groups, implicate that they likely perform diverse functions in supporting soybean normal growth and development.

### Expression patterns of *GmEIL* genes against abiotic stresses

**Figure 5:**
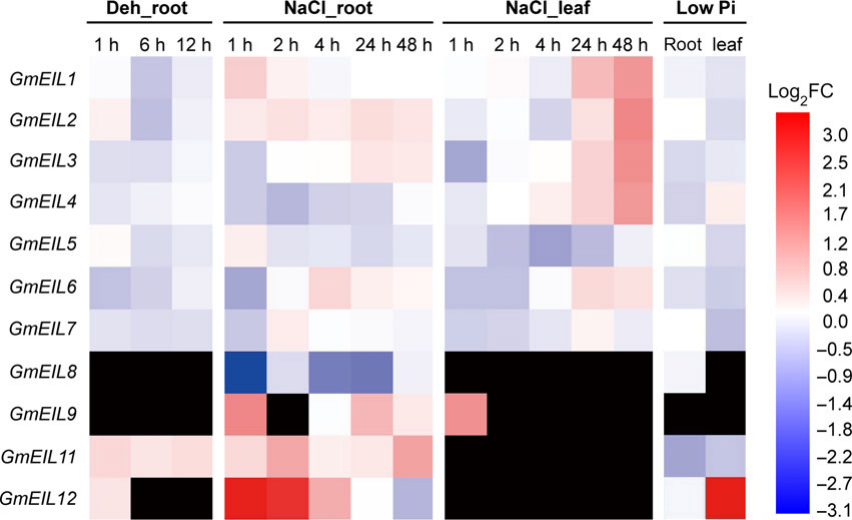
The expression patterns of *GmEIL* genes against abiotic stresses. Gradient colors represent log2 FC in gene expression of different samples compared with control.

Ethylene is regarded as a stress hormone involved in myriad stress responses. Several ethylene signaling components, including EIN3 and EIL1, have been shown to regulate plant stresses 1, 51. Among these stresses, soil drought and salinity are the two most common and serious abiotic stresses limiting plant growth and crop productivity. To explore the potential functions of *GmEIL* genes under these stresses, we detected their expression against dehydration and salinity (NaCl) treatments according to the previously released Illumina RNA‐seq data 40, 41. The result provided a basic impression of the expression changes of these genes except *GmEIL10*, which was not detected in almost all samples (Fig. 5). Under the dehydration condition, the transcripts of *GmEIL1* and *GmEIL2* were slightly decreased by about 0.7‐fold at 6 h compared with control (0 h) in the root. And the expression of *GmEIL6* was also moderately down‐regulated but earlier by 0.7‐fold at 1 h. In contrast, *GmEIL11* was slightly increased by about 1.5‐fold compared with the control. Under salt stress, the expression profiles of *GmEIL* genes were much more complicated; most of them were either positively or negatively regulated at least one time point *versus* control in root and leaf. For instance, *GmEIL2* and *GmEIL11* were increased, whereas *GmEIL4* and *GmEIL8* were decreased at almost every time point of NaCl treatment in root. Additionally, the expression of *GmEIL6* was down‐regulated at 1 h, but subsequently increased after a prolonged time of salt stress both in root and leaf. A similar expression pattern was observed for *GmEIL3* in leaf. In contrast, *GmEIL12* was obviously up‐regulated at the beginning of NaCl treatment, but moderately declined at 48 h of salt stress in root. Interestingly, *GmEIL1*,* GmEIL2*,* GmEIL3* and *GmEIL4* were obviously increased after 24 h of salt treatment in leaf.

Phosphorus (P) is an essential macronutrient for plant growth and development. Although P is abundant in most soils, phosphate (P_i_), the major form of P that plants assimilate, is limited. Thus, P is one of the most limiting nutrients for crop productivity 52. Increasing evidences indicate a key role for ethylene in regulating plant responses to P_i_ starvation 52, 53. Thus, we also analyzed the expression changes of *GmEIL* genes under P_i_ starvation using the published transcriptome data 42. As shown in Fig. 5, the expression of *GmEIL11* was down‐regulated both in root and in leaf. *GmEIL7* was slightly decreased by about 0.7‐fold in leaf, whereas *GmEIL12* was obviously up‐regulated by about 10.7‐fold in leaf.

### The genetic diversity of *GmEIL* genes in 302 resequenced soybean accessions

To study the allelic variations of *GmEIL* genes, we surveyed them in 302 resequenced soybean accessions, including 62 wild soybeans (*Glycine soja*), 130 landraces and 110 improved cultivars 44. On the whole, the number of non‐synonymous SNPs or non‐synonymous SNPs per kb CDS in *GmEIL* genes from group A was fewer than those from groups B and C, suggesting that these genes in group A are more conserved compared with those in groups B and C (Table 2). Furthermore, *GmEIL10* had the largest mean number of SNPs and non‐synonymous SNPs per kb sequence among these genes (Table 2), supporting that it is a potential pseudogene. It was noteworthy that only a few non‐synonymous SNPs were found at the conserved site, although some SNPs exist in these genes (Table 2).

**Table 2:** The SNP summary of *GmEIL* genes within 302 resequenced soybean accessions. SNP/kb: average number of SNPs per kb DNA sequence. NS SNP: non‐synonymous SNPs of each *EIL* gene in 302 soybean accessions. NS SNP/kb: mean number of non‐synonymous SNPs per kb CDS sequence

Gene	Total SNP	SNP/kb	NS SNP	NS SNP/kb	NS SNP at conserved site
*GmEIL1*	5	1.4	0	0	0
*GmEIL2*	7	2.2	2	1.1	1 in groups A, B, C
*GmEIL3*	25	7.3	4	2.2	1 in groups A, B
*GmEIL4*	21	6	0	0	0
*GmEIL5*	18	5.3	3	1.6	2 in group A
*GmEIL6*	36	12.1	6	3.4	0
*GmEIL7*	11	3.5	1	0.6	0
*GmEIL8*	113	9.7	7	5.1	2 in groups A, B, C
*GmEIL9*	7	5	6	4.3	0
*GmEIL10*	39	12.6	12	5.2	0
*GmEIL11*	22	9.5	6	4.3	1 in group C
*GmEIL12*	10	7.5	6	5	1 in groups A, B, C

Identification of genes associated with domestication and improvement is important for breeding superior varieties 44. To detect the potential selective signals during the processes of soybean domestication (wild soybeans *vs* landraces) and improvement (landraces *vs* improved cultivars), we compared the SNP distribution status of these genes in the aforementioned 302 soybean accessions. As a result, a total of 11 domestication‐selective non‐synonymous SNPs were identified, among which eight were equally distributed over *GmEIL6* and *GmEIL9* (Table S2). The remaining three domestication‐selective SNPs were in *GmEIL2* and *GmEIL12*. However, none of these domestication‐selective non‐synonymous SNPs occurred at the conserved site except one in *GmEIL2*. For *GmEIL2*, a domestication‐selective SNP (C→T, T corresponding the reference genome Wm82.a2.v1) was identified, which generates a missense mutation (R→C, C corresponding to the reference genome Wm82.a2.v1) at conserved 267 residues in predicted DNA binding domain BD IV (Table S2, Fig. S2). Association study of ethylene‐related agronomic traits and this allelic non‐synonymous mutation, phenotypic analysis of the transgenic soybean with two genotypes, and comparison of the biochemical properties of the two proteins will be useful to uncover the functional significance of this missense mutation.

## Discussion

EIN3 and EIL1 not only play a master role in the ethylene signaling transduction pathway, but also serve as a center that integrates ethylene with other signals, and thus broadly regulate plant growth and development as well as resistances to diverse stresses 4. Although the regulatory mechanisms of these genes are well illuminated in Arabidopsis, the molecular mechanisms in other plants remain obscure. Only a limited number of genome‐wide studies of the *EIL* family in plants have been previously reported, such as *Hevea brasiliensis* 54, Rosaceae 55 and poplar 56. In this study, the *EIL* TF family was comprehensively characterized in soybean, which provides more than half of global oilseed production and a quarter of the world's protein for human food and animal feed.

### The comparison of the *EIL* genes in soybean and Arabidopsis

A comparison of *EIL* homologs between Arabidopsis and soybean, including protein sequences and expression profiles, may provide valuable information to predict the potential functions of *GmEIL* genes. The present phylogenetic analysis showed that these EIL proteins were categorized into three clades (Fig. 2). Additionally, EIL members with similar gene structures and motif compositions clustered together, which was consistent with the EIL classification in other plants (Fig. 2) 55. The EILs that cluster together in the same group tend to possess similar functions. The *GmEIL* genes in group A (*GmEIL1* to *GmEIL5*) were the best orthology match of Arabidopsis *AtEIN3* and *AtEIL1*, implying their potential roles as primary positive regulators in the ethylene signaling pathway. And *GmEIL6* and *GmEIL7* from group B were the orthologs of *AtEIL3*, implicating that they might function like *AtEIL3* to regulate sulfur response and metabolism. The remaining *GmEIL* homologs in group C might have identical roles to their orthologs, *AtEIL4* and *AtEIL5*.

Gene expression patterns usually provide important clues relating to their functions. In general, soybean *GmEIL* genes displayed similar tissue expression patterns to those in Arabidopsis (Fig. 4, Fig. S5). Among them, *GmEIL* genes in group A and the orthologous *AtEIN3* and *AtEIL1* were preferentially expressed in almost every tissue. Conversely, these *EIL* genes from group C showed obviously lower or no expression in most samples. In addition, the remaining *EIL* genes were intermediately expressed in multiple tissues. It can be speculated that the variable spatio‐temporal expression patterns of soybean *GmEILs* may be related to their functional divergences. Further investigation using potential tools such as overexpression, antisense expression or mutant collection for altering the *GmEILs* expression levels will be helpful for infering their functions in soybean.

### *EIL* genes acted as the hub for modulating plant developmental and stress processes

*AtEIN3* and *AtEIL1* directly regulate a number of downstream transcriptional cascades, including a major feedback regulatory circuitry of the ethylene signaling pathway, and the orchestration of other hormone‐mediated growth response pathways 16. *AtEIL2* plays minor and partially redundant roles in the ethylene signaling pathway 18. On the contrary, *AtEIL3* is functionally distinct from other *EIL* family members mediating ethylene responses, and it is widely involved in the regulation of sulfur deficiency‐responsive genes that play essential roles in optimizing transport and internal utilization of sulfate in Arabidopsis 19. But how these genes are regulated remains unclear.

Preliminary stress‐related *cis*‐acting element analysis suggested that *EILs* might be key players mediating plant stress tolerances (Fig. S6). For example, 10 *EIL*s (*AtEIL1*,* AtEIL3*,* GmEIL2*,* GmEIL3*,* GmEIL4*,* GmEIL6*,* GmEIL8*,* GmEIL9*,* GmEIL11* and *GmEIL12*), four *EIL*s (*AtEIL1*,* AtEIL3*,* AtEIL5* and *GmEIL11*), 15 *EIL*s (*AtEIN3*,* AtEIL1*,* AtEIL2*,* AtEIL3*,* AtEIL4*,* AtEIL5*,* GmEIL3*,* GmEIL4*,* GmEIL5*,* GmEIL6*,* GmEIL7*,* GmEIL8*,* GmEIL10*,* GmEIL11* and *GmEIL12*) and seven *EIL*s (*AtEIL1*,* AtEIL5*,* GmEIL1*,* GmEIL2*,* GmEIL4*,* GmEIL7* and *GmEIL11*) appeared to be responsive to drought, cold, heat and fungal‐related stresses, respectively, since their promoter regions contained specific stress‐related *cis*‐elements. In this study, we found the expression of *GmEIL2*,* GmEIL6* and *GmEIL11* were slightly regulated against drought, whereas other *GmEIL* genes did not respond to drought (Fig. 5). This result is not exactly consistent with the predicted results acquired from the *cis*‐acting element analysis in their promoter regions. One reasonable explanation is that other factors, such as chromatin accessibility and additional cofactors, may play a more important role in regulating these *GmEILs*’ transcription than *trans*‐acting TFs that bind to *cis*‐regulatory elements in their promoters.

Hormonal signals control almost all the stages of growth and development by regulating gene expression, which in turn translates into appropriate morphological or physiological responses. The promoter *cis*‐element prediction revealed that different *EILs* possessed different hormone‐related elements (Fig. S6). Among them, auxin, gibberellin, abscisic acid, ethylene, methyl jasmonate and salicylic acid responsive elements were observed in five *EILs* (*AtEIL1*,* AtEIL2*,* AtEIL3*,* GmEIL5* and *GmEIL11*), 16 *EILs* (all *EILs* in Arabidopsis and soybean except *AtEIL4* and *GmEIL7*), eight *EILs* (*AtEIN3*,* AtEIL2*,* AtEIL4*,* AtEIL5*,* GmEIL4*,* GmEIL9*,* GmEIL11* and *GmEIL12*), seven *EILs* (*AtEIL4*,* AtEIL5*,* GmEIL3*,* GmEIL5*,* GmEIL6*,* GmEIL10* and *GmEIL12*), 11 *EILs* (*AtEIN3*,* AtEIL1*,* AtEIL2*,* AtEIL4*,* GmEIL1*,* GmEIL2*,* GmEIL3*,* GmEIL4*,* GmEIL5*,* GmEIL8* and *GmEIL12*) and 14 *EILs* (all *EILs* in Arabidopsis and soybean except *AtEIL4*,* GmEIL1*,* GmEIL7* and *GmEIL12*), respectively. Most promoters of these *EIL* genes included a combination of multiple hormone‐related elements. These data support that *EIL* genes play important roles in phytohormone signaling pathways, and implicate that *EIL* genes could be transcriptionally regulated by a variety of hormones. Alternatively, these *EIL* genes may require a post‐transcriptional regulation mechanism, since none of the genes *AtEIN3*,* AtEIL1* and *AtEIL3* is transcriptionally regulated in response to ethylene or sulfur. What is more, the protein levels of AtEIN3 and AtEIL1 are strictly regulated by ethylene through a post‐transcriptional mechanism 17, 19. Further associated analysis of the gene expression abundances under specific conditions and their promoter characteristics will validate whether the expression of *EIL* genes is regulated by the hormones and stresses.

### The conservation of *GmEIL* genes

The cultivated soybeans were domesticated from wild soybeans (*G. sojas*) in China about 5000 years ago. They were exported to Korea and Japan approximately 2000 years ago, to North America in 1765, and to Central and South America during the first half of the 20th century 44. Genetically, domestication is a process of modifying genome diversity in the cultivated varieties 57. It has suggested that there were several genetic bottlenecks during soybean domestication and improvement 58. Detection of genome‐wide genetic diversity and identification of genes relevant to domestication and improvement will be helpful for future crop improvement 44, 59. In this study, we investigated the allelic variations of *GmEIL* genes in 302 resequenced soybean accessions. Our data revealed that these *GmEIL* genes are well conserved, especially *GmEIL* genes from group A, since only a few non‐synonymous SNPs were discovered at the conserved site (Table 2). This result is consistent with the fact that these *GmEIL* genes were powerfully negative selected during evolution (Table S1). In our previous study, we identified a total of 121 domestication‐selective sweeps and 109 improvement‐selective sweeps using the 302 resequenced wild and cultivated accessions 44. By comparing the physical location of *GmEIL* genes in soybean genome, we found that none of these genes exists in the selective sweeps except *GmEIL1*. Although *GmEIL1* was found in a domestication‐selective sweep, it does not have any non‐synonymous SNPs (Table 2). What is more, although we identified 11 domestication‐selective non‐synonymous SNPs in *GmEIL* genes, they did not occur at the conserved site except for one in *GmEIL2* (Table S2). These results suggest that the *GmEIL* genes may not undergo selection during domestication and improvement. Their versatility and complexity as well as highly functional redundancy could explain why most *GmEIL* genes are neither domesticated nor improved.

In sum, 12 *GmEIL* genes were identified in the soybean genome. We comprehensively analyzed their basic physical and chemical properties, phylogenetic relationships, gene structures, motif compositions, duplication status, spatio‐temporal and stressed expression patterns, and genetic variations. These results contribute to further study of the function of *EIL* genes in soybean.

## Conflict of interest

The authors declare no conflict of interest.

## Author contributions

QL, CF and YZ conceived and designed research. YS mainly contributed to RNA‐seq analysis. LG, HW and YZ contributed to data collection. CF mainly responsible for collinear analysis. QL analyzed all the data. QL wrote the manuscript. CF and YZ contributed to revising the manuscript. All authors read and approved the manuscript.

## Supporting information


**Fig. S1.** The conserved domains of EIL proteins from Arabidopsis and soybean. Pfam program was used to identify the conserved domains of 18 EIL proteins.
**Fig. S2.** The alignment of EIL proteins from Arabidopsis and soybean. Red arrows indicate the mutation positions of *slim1‐1*,* slim1‐2*,* slim1‐3*,* slim1‐4* and *ein3‐3*. Green arrow shows the domesticated mutation site in GmEIL2. Predicted DNA binding domains (BD I to BD IV) were underlined.
**Fig. S3.** The sequence identity analysis of EIL proteins from Arabidopsis and soybean.
**Fig. S4.** The amino acid constitution of each motif in EIL proteins. Multilevel consensus sequences were predicted by meme tool.
**Fig. S5.** The spatio‐temporal expression patterns of *EIL* genes in Arabidopsis. The expression values were obtained from Tair. Gradient colors indicate log2 transformed expression values in different samples.
**Fig. S6.** The statistics of the *cis*‐acting elements in each promoter region of *EIL* genes. PlantCARE was used to identify the *cis*‐acting elements in the promoters (1.5 kb upstream of ATG site) of 18 *EIL* genes. Based on the functional annotations, the *cis*‐acting elements were divided into four major classes: development‐, hormone‐, stress‐, and light responsiveness‐related *cis*‐acting elements. The value shown here for the development or light responsiveness‐related *cis*‐acting elements is the total number of each element in this class. Gradient colors indicated log2 transformed values for the *cis*‐acting elements.

**Table S1.** The *K*_a_ and *K*_s_ values among *GmEIL* genes.

**Table S2.** The SNP distribution of *GmEIL* genes in 302 resequenced soybean accessions.
