# Supplementary material for: The EIL transcription factor family in soybean: Genome‐wide identification, expression profiling and genetic diversity analysis
Source: FEBS Open Bio. 2019 Feb 21;9(4):629–42. doi: 10.1002/2211-5463.12596 (PMC6443860; doi:10.1002/2211-5463.12596)
Supplement: Supplementary file 1 — Fig. S1. The conserved domains of EIL proteins from Arabidopsis and soybean. Pfam program was used to identify the conserved domains of 18 EIL proteins. Fig. S2. The alignment of EIL proteins from Arabidopsis and soybean. Red arrows indicate the mutation positions of slim1‐1, slim1‐2, slim1‐3, slim1‐4 and ein3‐3. Green arrow shows the domesticated mutation site in GmEIL2. Predicted DNA binding domains (BD I to BD IV) were underlined. Fig. S3. The sequence identity analysis of EIL proteins from Arabidopsis and soybean. Fig. S4. The amino acid constitution of each motif in EIL proteins. Multilevel consensus sequences were predicted by meme tool. Fig. S5. The spatio‐temporal expression patterns of EIL genes in Arabidopsis. The expression values were obtained from Tair. Gradient colors indicate log2 transformed expression values in different samples. Fig. S6. The statistics of the cis‐acting elements in each promoter region of EIL genes. PlantCARE was used to identify the cis‐acting elements in the promoters (1.5 kb upstream of ATG site) of 18 EIL genes. Based on the functional annotations, the cis‐acting elements were divided into four major classes: development‐, hormone‐, stress‐, and light responsiveness‐related cis‐acting elements. The value shown here for the development or light responsiveness‐related cis‐acting elements is the total number of each element in this class. Gradient colors indicated log2 transformed values for the cis‐acting elements. [file FEB4-9-629-s001.pptx]

## Slide 1
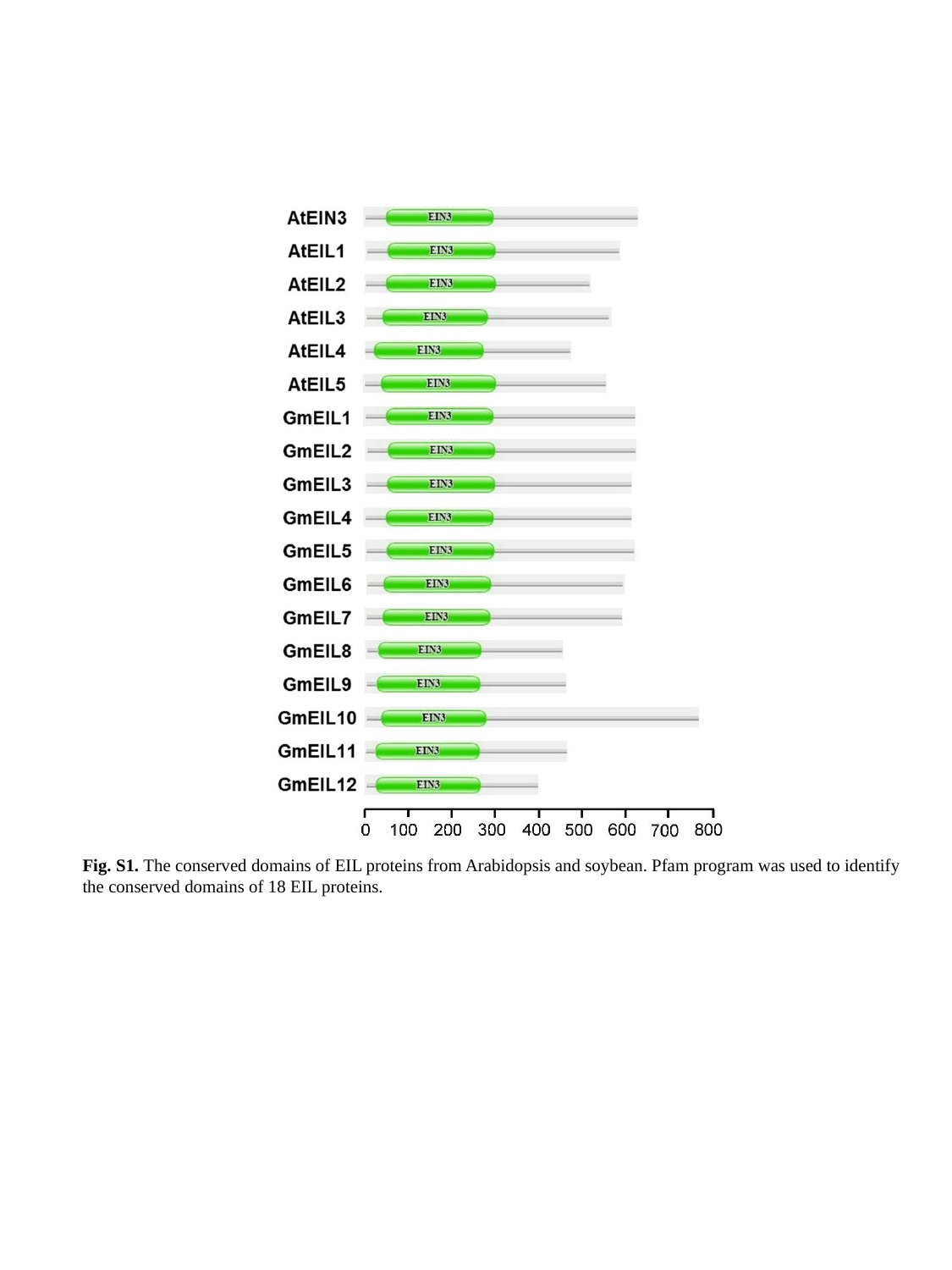

Fig. S1. The conserved domains of EIL proteins from Arabidopsis and soybean. Pfam program was used to identify the conserved domains of 18 EIL proteins.

## Slide 2
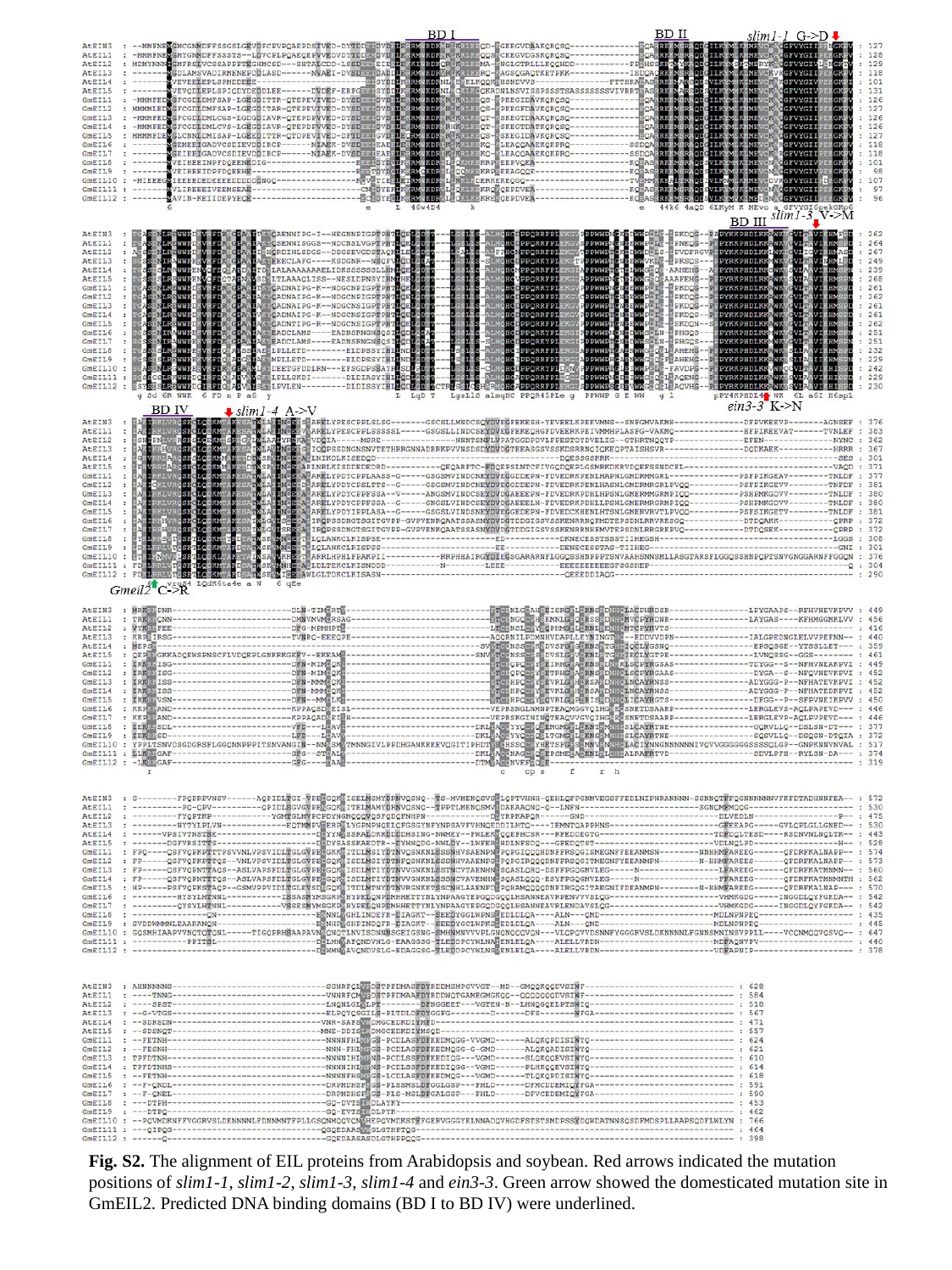

Fig. S2. The alignment of EIL proteins from Arabidopsis and soybean. Red arrows indicated the mutation positions of slim1-1, slim1-2, slim1-3, slim1-4 and ein3-3. Green arrow showed the domesticated mutation site in GmEIL2. Predicted DNA binding domains (BD I to BD IV) were underlined.

## Slide 3
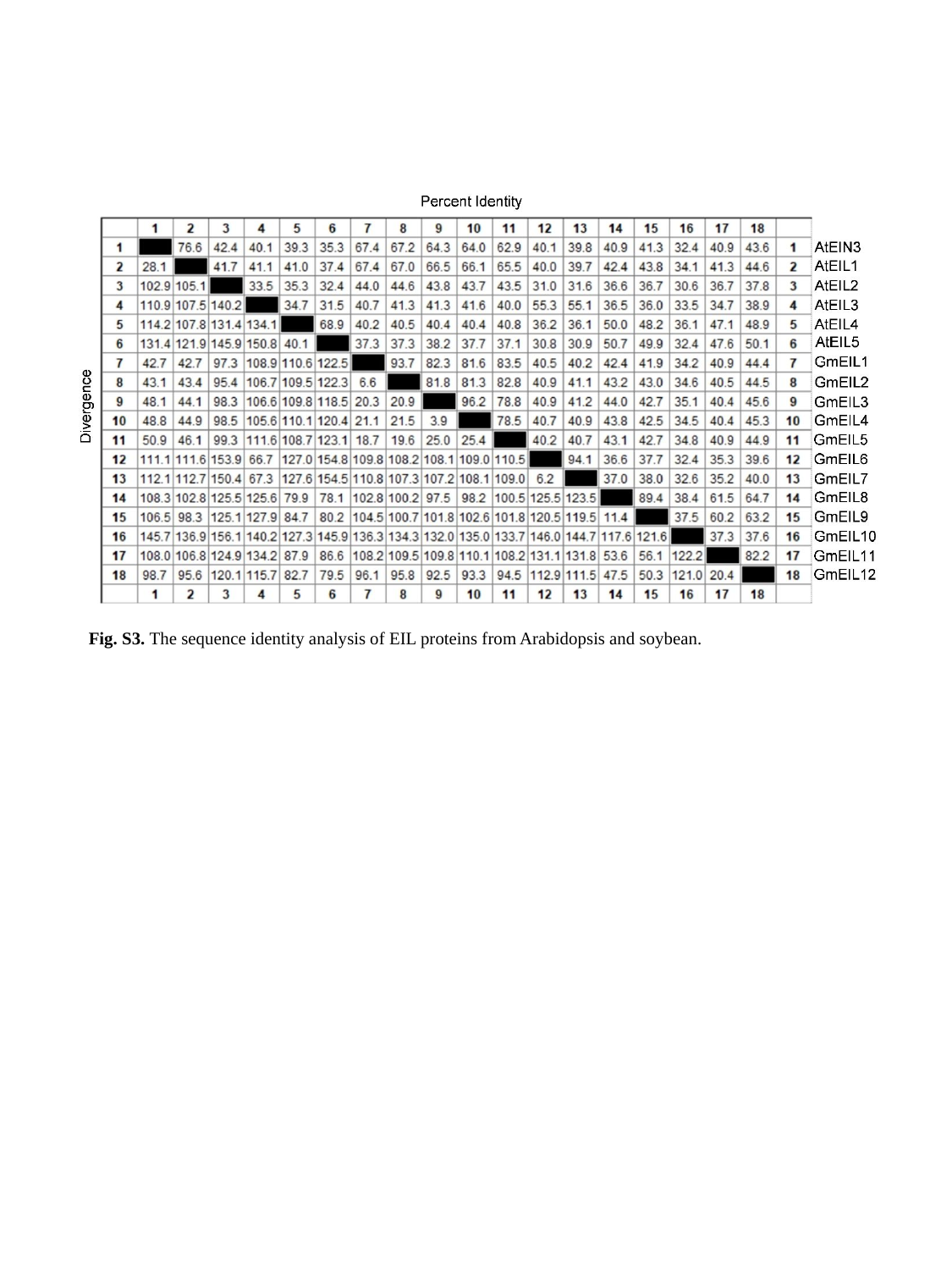

Fig. S3. The sequence identity analysis of EIL proteins from Arabidopsis and soybean.

## Slide 4
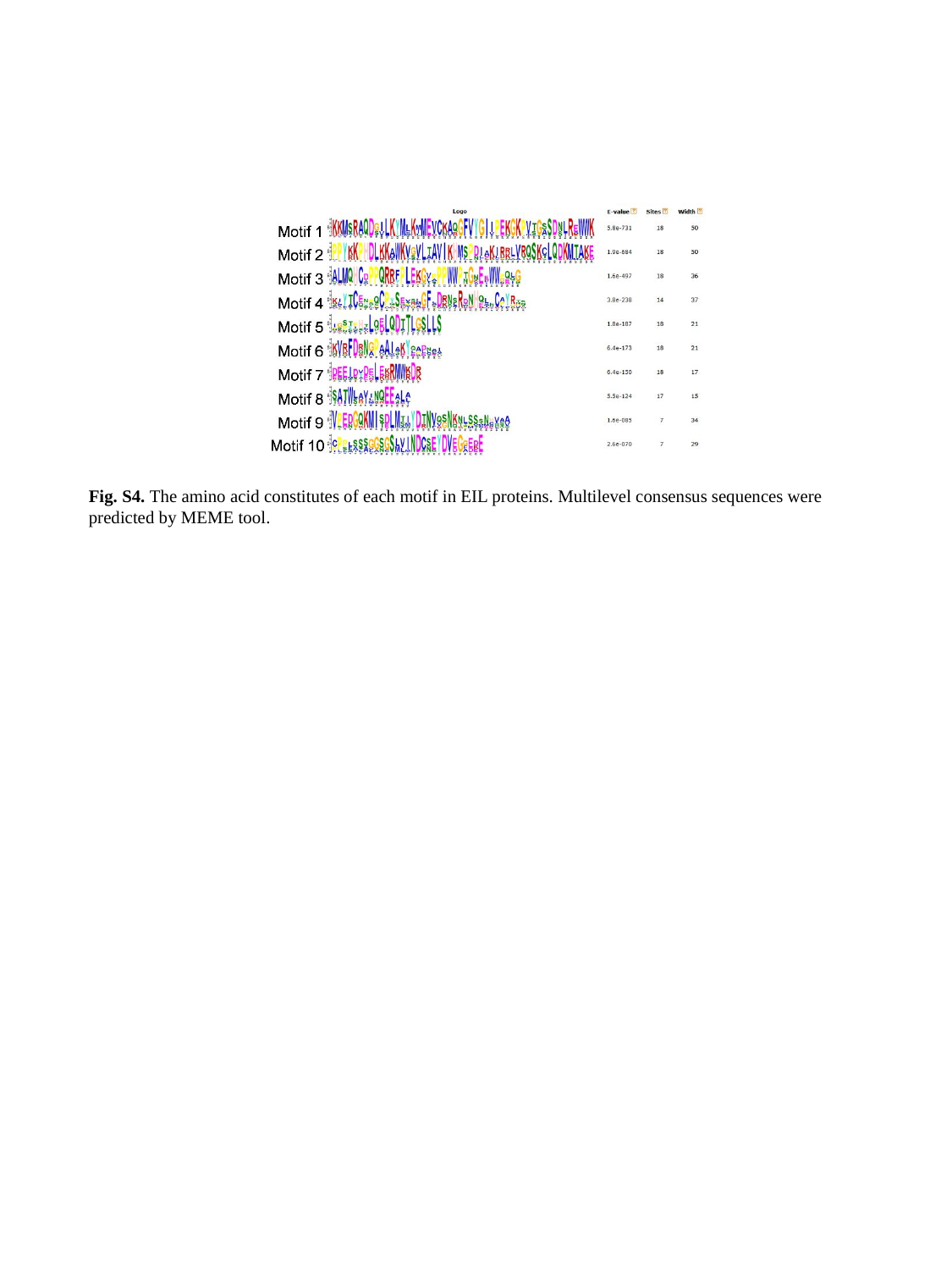

Fig. S4. The amino acid constitutes of each motif in EIL proteins. Multilevel consensus sequences were predicted by MEME tool.

## Slide 5
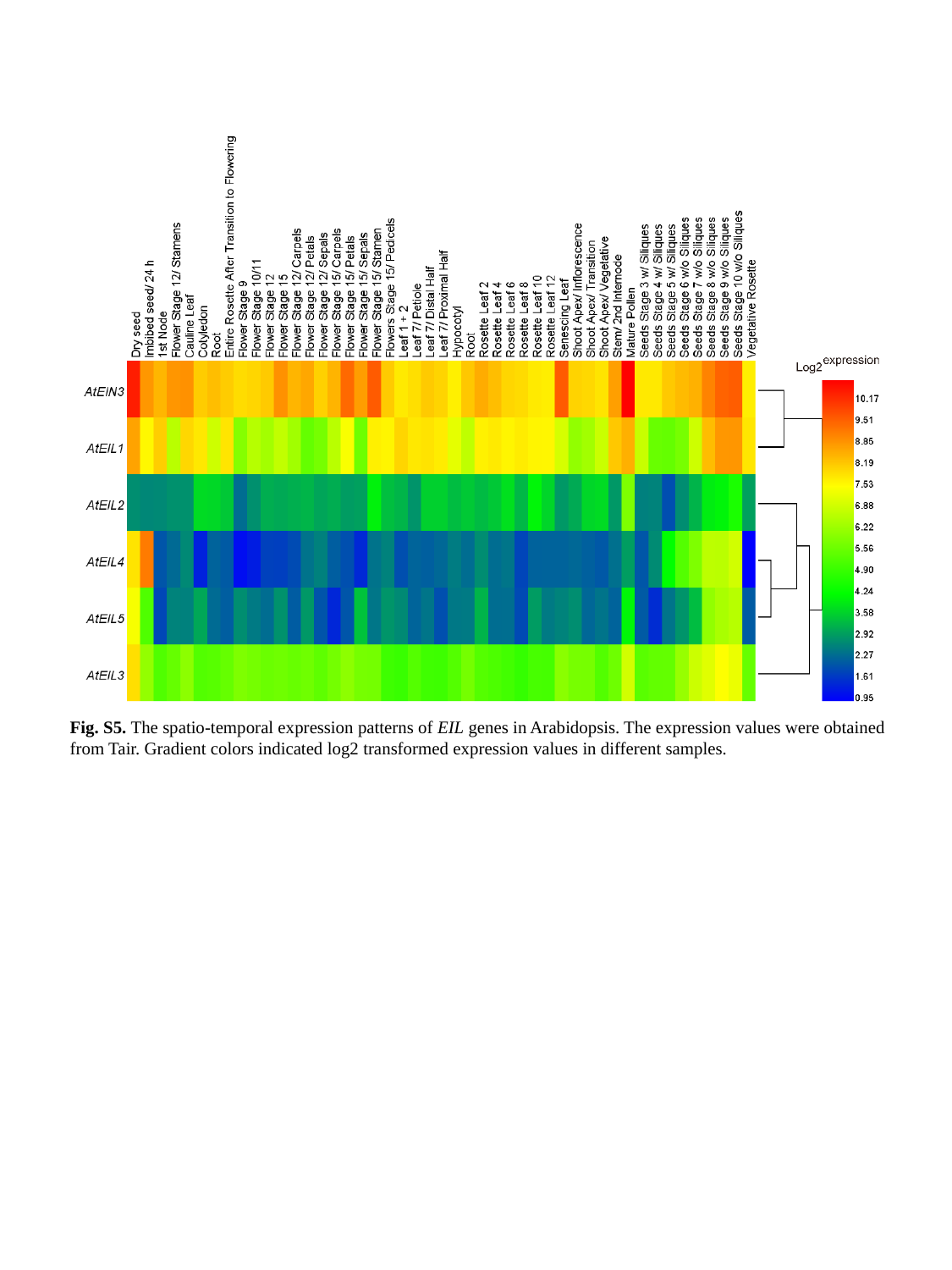

Fig. S5. The spatio-temporal expression patterns of EIL genes in Arabidopsis. The expression values were obtained from Tair. Gradient colors indicated log2 transformed expression values in different samples.

## Slide 6
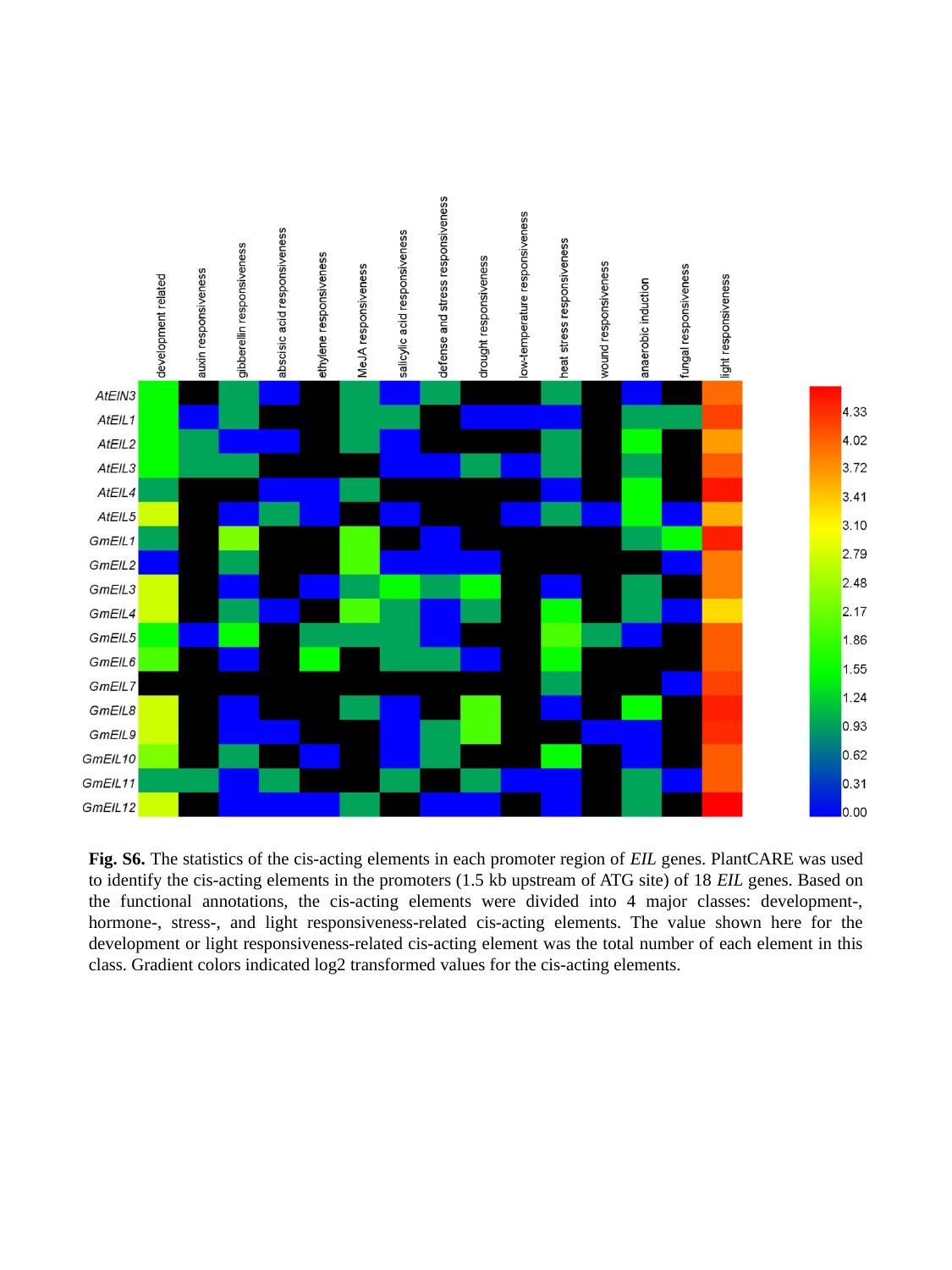

Fig. S6. The statistics of the cis-acting elements in each promoter region of EIL genes. PlantCARE was used to identify the cis-acting elements in the promoters (1.5 kb upstream of ATG site) of 18 EIL genes. Based on the functional annotations, the cis-acting elements were divided into 4 major classes: development-, hormone-, stress-, and light responsiveness-related cis-acting elements. The value shown here for the development or light responsiveness-related cis-acting element was the total number of each element in this class. Gradient colors indicated log2 transformed values for the cis-acting elements.
