# Supplementary material for: The EIL transcription factor family in soybean: Genome‐wide identification, expression profiling and genetic diversity analysis
Source: FEBS Open Bio. 2019 Feb 21;9(4):629–42. doi: 10.1002/2211-5463.12596 (PMC6443860; doi:10.1002/2211-5463.12596)
Supplement: Supplementary file 2 — Table S1. The K a and K s values among GmEIL genes. [file FEB4-9-629-s002.docx]

**Table S1.** The Ka and Ks values among *GmEIL* genes.

| **Locus 1** | **Locus 2** | **Ka** | **Ks** |  | **Ka/Ks** | **Duplication type** |
| --- | --- | --- | --- | --- | --- | --- |
| *GmEIL1* | *GmEIL2* | 0.0297 | 0.2122 |  | 0.14 | Glycine WGD |
| *GmEIL1* | *GmEIL4* | 0.1081 | 0.7865 |  | 0.14 | Legume WGD |
| *GmEIL3* | *GmEIL4* | 0.0161 | 0.1572 |  | 0.1 | Glycine WGD |
| *GmEIL6* | *GmEIL7* | 0.026 | 0.1256 |  | 0.21 | Glycine WGD |
| *GmEIL8* | *GmEIL11* | 0.2947 | 1.3133 |  | 0.22 | Legume WGD |
| *GmEIL8* | *GmEIL12* | 0.2801 | 1.3277 |  | 0.21 | Legume WGD |
| *GmEIL9* | *GmEIL11* | 0.3049 | 1.1154 |  | 0.27 | Legume WGD |
| *GmEIL9* | *GmEIL12* | 0.2499 | 1.4854 |  | 0.17 | Legume WGD |
| *GmEIL11* | *GmEIL12* | 0.1019 | 0.3785 |  | 0.27 | Glycine WGD |
