# Supplementary material for: The EIL transcription factor family in soybean: Genome‐wide identification, expression profiling and genetic diversity analysis
Source: FEBS Open Bio. 2019 Feb 21;9(4):629–42. doi: 10.1002/2211-5463.12596 (PMC6443860; doi:10.1002/2211-5463.12596)
Supplement: Supplementary file 3 — Table S2. The SNP distribution of GmEIL genes in 302 resequenced soybean accessions. [file FEB4-9-629-s003.docx]

**Table S2.** The SNP distribution of *GmEIL* genes in resequenced 302 soybean accessions.

|  | | | | | | | **Distribution frequency (%)** | | | | | | | | |
| --- | --- | --- | --- | --- | --- | --- | --- | --- | --- | --- | --- | --- | --- | --- | --- |
|  |  |  |  |  |  |  | **Elite cultivar** | | | **Landrace** | | | ***G. soja*** | | |
| **ID** | **SNP** | **Direction** | **SNP in genome** | **SNP location in gene** | **Mutation** | **Amino acid conservation** | **WT (Wm 82)** | **Heterozygous** | **Mutation** | **WT (Wm 82)** | **Heterozygous** | **Mutation** | **WT (Wm 82)** | **Heterozygous** | **Mutation** |
| *GmEIL2* | T/C | reverse | 18123516 | CDS 1 | N->D | not conserved | 100 | 0 | 0 | 100 | 0 | 0 | 88.7 | 3.2 | 8.1 |
| *GmEIL2* | A/G | reverse | 18124425 | CDS 1 | C->R | conserved in groups A, B, C | 65.5 | 5.5 | 29.1 | 76.2 | 3.8 | 20 | 1.6 | 3.2 | 95.2 |
| *GmEIL3* | T/G | forward | 3129595 | CDS 1 | D->E | conserved in groups A and B | 100 | 0 | 0 | 100 | 0 | 0 | 93.5 | 0 | 6.5 |
| *GmEIL3* | G/A | forward | 3130352 | CDS 1 | E->K | not conserved | 100 | 0 | 0 | 90.8 | 4.6 | 4.6 | 93.5 | 4.8 | 1.6 |
| *GmEIL3* | T/C | forward | 3130751 | CDS 1 | F->L | not conserved | 100 | 0 | 0 | 100 | 0 | 0 | 91.9 | 3.2 | 4.8 |
| *GmEIL3* | C/A | forward | 3130952 | CDS 1 | Q->K | not conserved | 86.4 | 3.6 | 10 | 72.3 | 3.1 | 24.6 | 77.4 | 9.7 | 12.9 |
| *GmEIL5* | G/A | forward | 18151054 | CDS 1 | E->K | conserved in group A | 100 | 0 | 0 | 96.9 | 0 | 3.1 | 90.3 | 4.8 | 4.8 |
| *GmEIL5* | G/A | forward | 18152275 | CDS 1 | G->S | conserved in group A | 100 | 0 | 0 | 100 | 0 | 0 | 91.9 | 1.6 | 6.5 |
| *GmEIL5* | A/C | forward | 18152690 | CDS 1 | H->P | not conserved | 100 | 0 | 0 | 100 | 0 | 0 | 93.5 | 0 | 6.5 |
| *GmEIL6* | G/A | reverse | 43397064 | CDS 2 | P->L | not conserved | 92.7 | 3.6 | 3.6 | 81.5 | 6.2 | 12.3 | 3.2 | 11.3 | 85.5 |
| *GmEIL6* | G/C | reverse | 43397166 | CDS 2 | P->R | not conserved | 94.5 | 3.6 | 1.8 | 83.8 | 3.1 | 13.1 | 50 | 11.3 | 38.7 |
| *GmEIL6* | C/T | reverse | 43397340 | CDS 2 | R->K | not conserved | 94.5 | 1.8 | 3.6 | 82.3 | 3.8 | 13.8 | 29 | 11.3 | 59.7 |
| *GmEIL6* | T/C | reverse | 43397427 | CDS 2 | Q->R | not conserved | 95.5 | 0.9 | 3.6 | 79.2 | 0 | 13.1 | 0 | 1.6 | 98.4 |
| *GmEIL6* | C/G | reverse | 43398708 | CDS 1 | E->Q | not conserved | 87.3 | 12.7 | 0 | 80 | 20 | 0 | 98.4 | 1.6 | 0 |
| *GmEIL6* | C/G | reverse | 43398712 | CDS 1 | M->I | not conserved | 81.8 | 18.2 | 0 | 65.4 | 34.6 | 0 | 38.7 | 61.3 | 0 |
| *GmEIL7* | G/C | forward | 2562402 | CDS 2 | A->P | not conserved | 74.5 | 5.5 | 20 | 76.9 | 2.3 | 20.8 | 88.7 | 1.6 | 9.7 |
| *GmEIL8* | G/A | forward | 10575914 | CDS 1 | V->M | not conserved | 80 | 7.3 | 12.7 | 84.6 | 3.1 | 12.3 | 98.4 | 1.6 | 0 |
| *GmEIL8* | A/G | forward | 10575956 | CDS 1 | N->D | not conserved | 100 | 0 | 0 | 100 | 0 | 0 | 88.7 | 4.8 | 6.5 |
| *GmEIL8* | C/G | forward | 10576208 | CDS 1 | P->A | conserved in groups A, B, C | 100 | 0 | 0 | 100 | 0 | 0 | 91.9 | 3.2 | 4.8 |
| *GmEIL8* | C/G | forward | 10576314 | CDS 1 | T->R | not conserved | 80 | 5.5 | 14.5 | 83.8 | 3.8 | 12.3 | 83.9 | 1.6 | 14.5 |
| *GmEIL8* | C/A | forward | 10576677 | CDS 1 | A->E | conserved in groups A, B, C | 100 | 0 | 0 | 99.2 | 0.8 | 0 | 93.5 | 1.6 | 4.8 |
| *GmEIL8* | C/T | forward | 10576748 | CDS 1 | P->S | not conserved | 100 | 0 | 0 | 100 | 0 | 0 | 85.5 | 0 | 14.5 |
| *GmEIL8* | G/A | forward | 10577007 | CDS 1 | R->H | not conserved | 86.4 | 2.7 | 10.9 | 60.8 | 9.2 | 30 | 79 | 4.8 | 16.1 |
| *GmEIL9* | G/T | forward | 36841823 | CDS 1 | L->F | not conserved | 99.1 | 0 | 0.9 | 98.5 | 0 | 1.5 | 82.3 | 4.8 | 12.9 |
| *GmEIL9* | C/G | forward | 36842343 | CDS 1 | L->V | not conserved | 84.5 | 4.5 | 10.9 | 52.3 | 8.5 | 39.2 | 17.7 | 11.3 | 71 |
| *GmEIL9* | C/T | forward | 36842413 | CDS 1 | T->M | not conserved | 85.5 | 3.6 | 10.9 | 53.1 | 0 | 39.2 | 14.5 | 11.3 | 74.2 |
| *GmEIL9* | A/G | forward | 36842470 | CDS 1 | Y->C | not conserved | 100 | 0 | 0 | 100 | 0 | 0 | 90.3 | 1.6 | 8.1 |
| *GmEIL9* | T/A | forward | 36842494 | CDS 1 | V->D | not conserved | 86.4 | 2.7 | 10.9 | 53.1 | 7.7 | 39.2 | 1.6 | 1.6 | 96.8 |
| *GmEIL9* | C/T | forward | 36842566 | CDS 1 | A->V | not conserved | 86.4 | 2.7 | 10.9 | 53.1 | 10 | 36.9 | 4.8 | 8.1 | 87.1 |
| *GmEIL10* | G/T | reverse | 50290260 | CDS 3 | Q->K | not conserved | 78.2 | 21.8 | 0 | 60 | 40 | 0 | 59.7 | 40.3 | 0 |
| *GmEIL10* | C/T | reverse | 50290292 | CDS 3 | A->T | not conserved | 72.7 | 27.3 | 0 | 60 | 40 | 0 | 59.7 | 40.3 | 0 |
| *GmEIL10* | C/T | reverse | 50290433 | CDS 3 | V->I | not conserved | 87.3 | 12.7 | 0 | 83.1 | 16.9 | 0 | 85.5 | 14.5 | 0 |
| *GmEIL10* | C/A | reverse | 50290434 | CDS 3 | R->S | not conserved | 89.1 | 9.1 | 1.8 | 100 | 0 | 0 | 93.5 | 6.5 | 0 |
| *GmEIL10* | A/G | reverse | 50290513 | CDS 3 | L->P | not conserved | 98.2 | 0.9 | 0.9 | 89.2 | 1.5 | 9.2 | 98.4 | 1.6 | 0 |
| *GmEIL10* | A/T | reverse | 50290648 | CDS 3 | V->E | not conserved | 100 | 0 | 0 | 100 | 0 | 0 | 88.7 | 4.8 | 6.5 |
| *GmEIL10* | T/A | reverse | 50290711 | CDS 3 | K->M | not conserved | 100 | 0 | 0 | 100 | 0 | 0 | 91.9 | 4.8 | 3.2 |
| *GmEIL10* | C/T | reverse | 50291171 | CDS 3 | G->R | not conserved | 89.1 | 5.5 | 5.5 | 100 | 0 | 0 | 91.9 | 4.8 | 3.2 |
| *GmEIL10* | C/T | reverse | 50291390 | CDS 3 | G->S | not conserved | 100 | 0 | 0 | 99.2 | 0.8 | 0 | 88.7 | 4.8 | 6.5 |
| *GmEIL10* | G/C | reverse | 50291659 | CDS 3 | A->G | not conserved | 50 | 29.1 | 20.9 | 56.2 | 26.2 | 17.7 | 53.2 | 19.4 | 27.4 |
| *GmEIL10* | G/T | reverse | 50293164 | CDS 1 | Q->k | not conserved | 69.1 | 10.9 | 19.1 | 86.2 | 6.2 | 7.7 | 98.4 | 1.6 | 0 |
| *GmEIL10* | G/T | reverse | 50293260 | CDS 1 | Q->K | not conserved | 53.6 | 10 | 36.4 | 36.2 | 6.9 | 56.9 | 50 | 11.3 | 38.7 |
| *GmEIL11* | C/T | forward | 1347269 | CDS 1 | A->V | conserved in group C | 100 | 0 | 0 | 100 | 0 | 0 | 93.5 | 1.6 | 4.8 |
| *GmEIL11* | T/A | forward | 1347455 | CDS 1 | L->H | not conserved | 86.4 | 13.6 | 0 | 95.4 | 4.6 | 0 | 93.5 | 6.5 | 0 |
| *GmEIL11* | G/A | forward | 1347497 | CDS 1 | G->E | not conserved | 90 | 10 | 0 | 91.5 | 8.5 | 0 | 93.5 | 6.5 | 0 |
| *GmEIL11* | C/T | forward | 1347670 | CDS 1 | R->C | not conserved | 22.7 | 4.5 | 72.7 | 14.6 | 3.8 | 81.5 | 1.6 | 0 | 98.4 |
| *GmEIL11* | T/C | forward | 1347929 | CDS 1 | V->A | not conserved | 39.1 | 10.9 | 50 | 27.7 | 8.5 | 63.8 | 22.6 | 6.5 | 71 |
| *GmEIL11* | G/T | forward | 1347950 | CDS 1 | G->V | not conserved | 100 | 0 | 0 | 99.2 | 0.8 | 0 | 91.9 | 1.6 | 6.5 |
| *GmEIL12* | T/G | reverse | 33338116 | CDS 1 | D->A | not conserved | 100 | 0 | 0 | 100 | 0 | 0 | 90.3 | 3.2 | 6.5 |
| *GmEIL12* | T/C | reverse | 33338486 | CDS 1 | K->E | conserved in groups A, B, C | 100 | 0 | 0 | 100 | 0 | 0 | 80.6 | 4.8 | 14.5 |
| *GmEIL12* | A/T | reverse | 33338527 | CDS 1 | V->E | not conserved | 55.5 | 15.5 | 29.1 | 36.9 | 7.7 | 55.4 | 4.8 | 6.5 | 88.7 |
| *GmEIL12* | T/A | reverse | 33338713 | CDS 1 | Y->F | not conserved | 60.9 | 12.7 | 26.4 | 43.1 | 7.7 | 49.2 | 62.9 | 12.9 | 24.2 |
| *GmEIL12* | C/G | reverse | 33338744 | CDS 1 | V->L | not conserved | 60 | 12.7 | 27.3 | 43.1 | 7.7 | 49.2 | 61.3 | 12.9 | 25.8 |
| *GmEIL12* | G/A | reverse | 33339118 | CDS 1 | A->V | not conserved | 69.1 | 7.3 | 23.6 | 43.1 | 7.7 | 49.2 | 12.9 | 11.3 | 75.8 |

Note: The domesticated SNPs are shown in red.
